# A unique three-way Philadelphia chromosome variant t(4;9;22)(q21;q34;q11.2) in a newly diagnosed patient with chronic phase chronic myeloid leukemia: a case report and review of the literature

**DOI:** 10.1186/s13256-021-02885-4

**Published:** 2021-05-25

**Authors:** Yuka Torii, Kana Nanjo, Tomomi Toubai, Masashi Hosokawa, Ryo Sato, Akane Yamada, Keiko Aizawa, Masahito Himuro, Satoshi Ito, Masakazu Yamamoto, John Magenau, Ryan Wilcox, Kenichi Ishizawa

**Affiliations:** 1 Department of Internal Medicine III, Division of Hematology and Cell Therapy, Yamagata University faculty of Medicine, 2-2-2 Iida-Nishi, Yamagata, 990-9585 Japan; 2 Faculty of Medicine, Yamagata University, Yamagata, Japan; 3 Department of Internal Medicine, Division of Hematology and Oncology, University of Michigan Medical School, Ann Arbor, MI USA

**Keywords:** t(4;9;22)(q21;Q34; Q11.2), CML, Philadelphia chromosome, Three-way variant

## Abstract

**Background:**

Chronic myeloid leukemia is a hematologic malignancy associated with the fusion of two genes: BCR and ABL1. This fusion results from a translocation between chromosomes 9 and 22, which is called the Philadelphia chromosome. Although the Philadelphia chromosome is present in more than 90% of patients with chronic myeloid leukemia, 5–8% of patients with chronic myeloid leukemia show complex variant translocations. Herein, we report a unique case of a three-way translocation variant in chronic phase chronic myeloid leukemia.

**Case presentation:**

A 40-year-old Asian male who presented with leukocytosis was diagnosed with chronic phase chronic myeloid leukemia. Cytogenetic karyotyping analysis showed 46,XY,t(4;9;22)(q21;q34;q11.2). He was treated with bosutinib and then changed to dasatinib because of intolerance, and MR4.5 (BCR-ABL/ABL ≦ 0.0032%, international scale) was achieved after 17 months of continuous treatment.

**Conclusion:**

This was the 14th case of t(4;9;22), in particular, a new variant Ph translocation involved in chromosome 4q21 and the first successful case treated with tyrosine kinase inhibitors in the world. We summarize previous case reports regarding three-way variant chromosome translocation, t(4;9;22) and discuss how this rare translocation is linked to prognosis.

## Introduction

Chronic myeloid leukemia (CML) is a myeloproliferative neoplasm characterized by the dysregulated production and uncontrolled proliferation of mature granulocytes with normal differentiation. CML is associated with the BCR-ABL1 fusion gene that results from a translocation between chromosomes 9 and 22, t(9;22)(q34;q11), called the Philadelphia (Ph) chromosome [1]. BCR-ABL1 is capable of autophosphorylation and uncontrolled signaling to multiple downstream oncogenic proteins in CML [2]. Tyrosine kinase inhibitors (TKIs) inhibit the initiation of the BCR-ABL1 pathway and are effective, frontline therapies for chronic phase (CP) CML (CML-CP) [3].

Ph chromosome is present in more than 90% of CML patients, and only about 5% of CML patients show complex variant translocations, which is due to the participation of one or more chromosomes other than 9 and 22 [4]. The mechanisms of the generation of the variant translocations are not fully understood. While some previous studies have suggested that CML patients with variant Ph translocations may have a worse outcome than those with classic translocations, other studies have shown that patients with variant Ph translocations have an outcome similar to those with classic Ph translocations when treated with imatinib mesylate [4, 5].

Herein, we describe a unique case of CML-CP with a three-way Ph chromosome variant t(4;9;22)(q21;q34;q11.2). This was the 14th case of t(4;9;22), in particular, a new variant Ph translocation involved in chromosome 4q21 and the first case treated with TKIs. In addition, we summarize previous case reports regarding three-way variant chromosome translocation t(4;9;22) and discuss how this rare translocation is linked to pathogenesis, disease characteristics, treatment responses, and prognosis.

## Case presentation

**Fig. 1:**
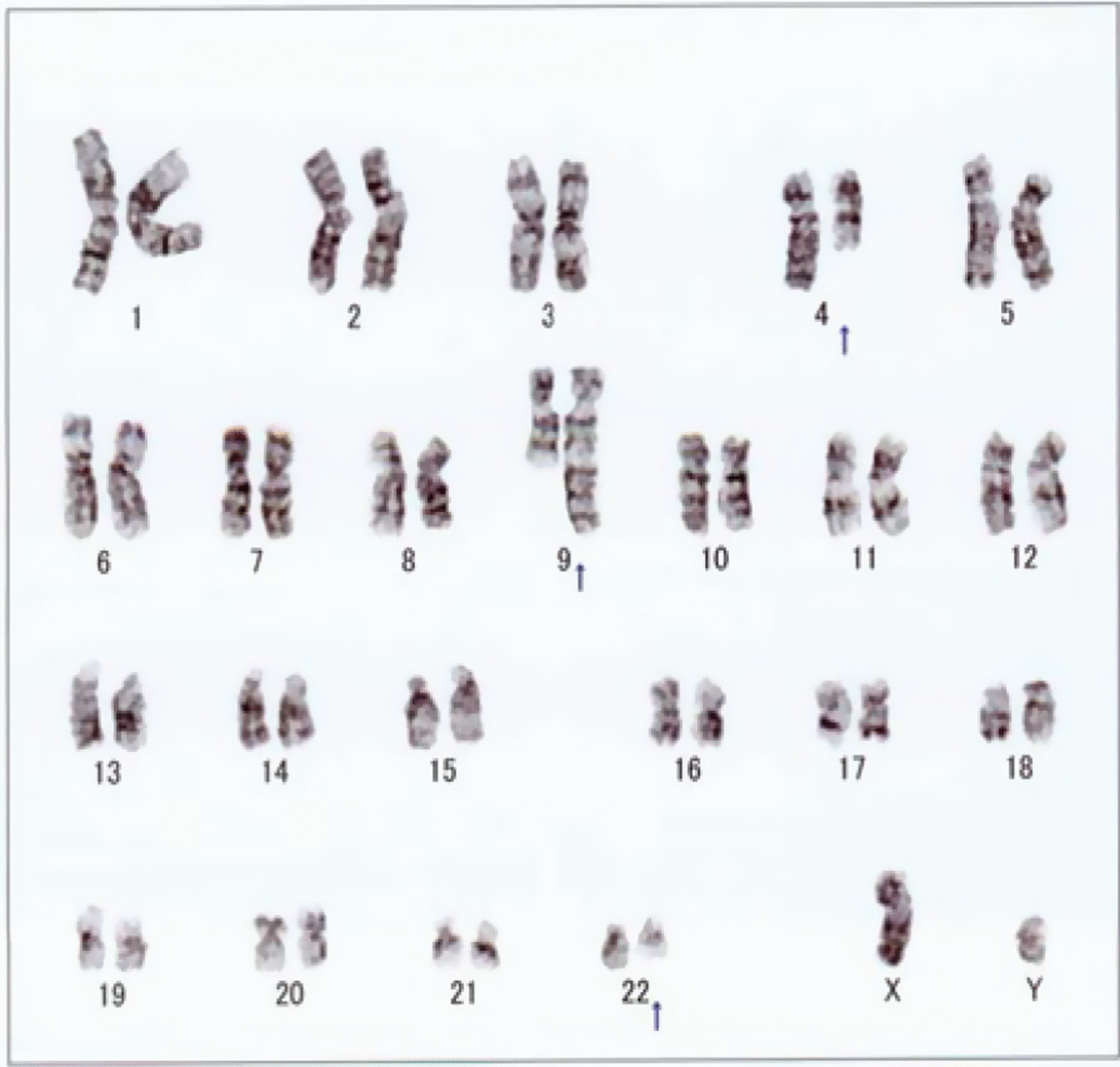
Cytogenetic analysis showing a variant three-way translocation: 46, XY, t(4;9;22)(q21;q34;q11.2). Arrowheads indicate all the derivative chromosomes

A 40-year-old Asian male with leukocytosis visited our hospital in June 2017. His physical examination was unremarkable, and no hepatosplenomegaly was observed. Hematological analysis revealed a white blood cell (WBC) count of 89.34 × 10^9^/l, consisting of 67.0% neutrophils, 5.5% lymphocytes, 0.5% monocytes, 0.5% eosinophils, 3.0% basophils, 1.5% promyelocytes, 14.5% myelocytes, and 7.5% metamyelocytes, a hemoglobin level of 12.6 g/dl, and platelet count of 381 × 10^9^/l. The bone marrow aspirate showed hypercellular bone marrow with 10.2% erythroid precursors, 0.2% myeloid blasts, 4.8% promyelocytes, 23.0% myelocytes, 17.0% metamyelocytes, 33.0% neutrophils, 8.2% eosinophils, 1.4% basophils, 1.8% lymphocytes, and 0.4% monocytes. Cytogenetic analysis was performed utilizing bone marrow culture cells, and all analyzed cells showed a complex, three-way (4;9;22)(q21;q34;q11.2) Ph chromosome translocation (Fig. 1). Therefore, he was diagnosed with CML-CP, and his Sokal and EUTOS long-term survival (ELTS) score were 0.586 (low risk) and 0.8248 (low risk), respectively.

**Fig. 2:**
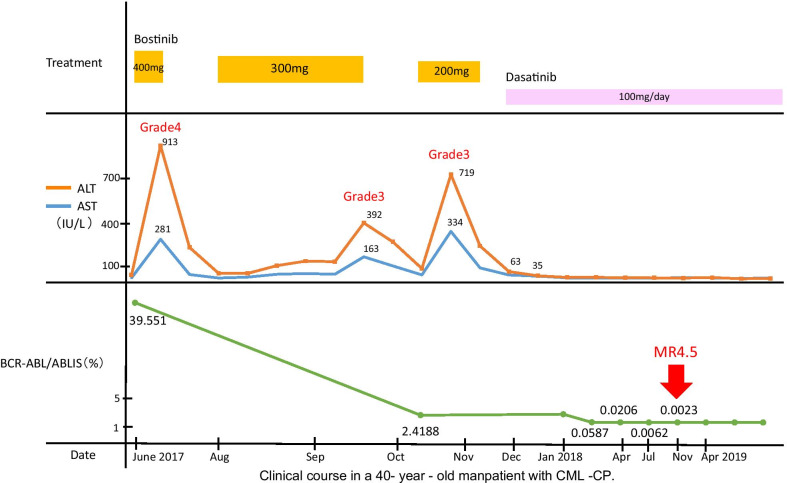
Clinical course in a 40-year-old man patient with CML-CP

Initially, he was treated with orally administered bosutinib at a daily dose of 400 mg. However, he had transient, grade 3 or 4 elevations in liver transaminases during treatment. Therefore, bosutinib was discontinued, and dasatinib at a daily dose of 100 mg was administered in December 2017. His BCR-ABL/ABL levels at 3, 6, and 12 months were 2.4188%, 2.7149%, and 0.0062%, respectively. Seventeen months after initial treatment, MR4.5 [BCR-ABL/ABL ≦ 0.0032% international scale (IS)] was achieved, and complete molecular response (CMR) was confirmed in April 2019 (Fig 2).

### Literature review and discussion

Ph chromosome is present in more than 90% of CML patients, and only about 5–8% of CML patients show complex variant translocations due to the participation of one or more chromosomes other than chromosomes 9 and 22 [4–7]. Fabarius *et al.* analyzed 1151 patients with Ph-chromosome-positive CML, and 69 patients (6.0%) had variant Ph translocations. Fifty two patients had three-way variant Ph translocation, and the frequency of the third partner chromosome for the three-way Ph variant translocation was 6/52 for chromosome 2 and 6/52 for chromosome 15. Only 2 patients in these 52 patients with three-way variant Ph translocation had t(4:9:22) [6].

On literature review, we found that t(4;9;22) as complex variant translocations has been reported in only 13 cases of CML [6, 8–15]. Table 1 presents a summary. In these cases, 4p16 and 4q25 have been reported as the major breakpoints on chromosome 4. However, the breakpoint at 4q21 is novel. Potentially relevant genes located on chromosome 4q21 are protein tyrosine-phosphatase non-receptor type 13 (PTPN13) [16], chemokine (C-X-C motif) ligand (CXCL)9–11 [17], RAP1 GTPase-GDP dissociation stimulator 1 (RAP1GDS1) [18], and ALL1-fused gene from chromosome 4.

**Table 1.** Summary of CML patients with t(4;9;22) in previous reports

Case	Translocations	Breakpoint	Age (years)	Sex	Phase at diagnosis	WBC	Treatment	Reference
1	t(4;9;22)(q28;q34;q11)	4q28	17	F	CP	–	–	[6]
2	t(4;9;22)(p15;q34;q11)	4p15	58	M	CP	–	–	[6]
3	t(4;9;22)(p16;q34;q11)	4p16	12	M	BC	–	–	[8]
4	t(4;9;22)(p16;q34;q11)	4p16	78	M	CP	236× ${10}^{9}$/l	HU	[9]
5	t(4;9;22)(p16;q34;q11)	4p16	42	M	CP	–	–	[10]
6	t(4;9;22)(p16;q34;q11)	4p16	59	M	CP	300× ${10}^{9}$/l	HU	[11]
7	t(4;9;22)(p16;q34;q11)	4p16	–	F	–	–	–	[12]
8	t(4;9;22)(p14;q34;q11)	4p14	–	M	–	–	–	[12]
9	t(4;9;22)(q25;q34;q11)	4q25	42	M	AP	72.8× ${10}^{9}$/l	IFN-α (9 months)	[13]
10	t(4;9;22)(q25;q34;q11)	4q25	–	–	CP	–	HU, chemotherapy	[13]
11	t(4;9;22)(p16;q34;q11)	4p16	–	–	–	–	–	[14]
12	t(4;9;22)(p16;q34;q11)	4p16	–	–	–	–	–	[14]
13	t(4;9;22)(q12;q34;q11)	4q12	44	M	CP	–	BMT	[15]
14	t(4;9;22)(q21;q34;q11.9)	4q21	40	M	CP	89.3× ${10}^{9}$/l	TKIs	Present case

*BC* blast crisis, *CP* chronic phase, *AP* accelerated phase, *HU* hydroxyurea, *IFN* interferon, *BMT* bone marrow transplantation, *TKI* tyrosine kinase inhibitor

AF4)[19] . The PTPN13 gene encodes the PTPN13, which is a negative regulator of tumor growth in human breast and ovarian cancer [20], but its pathological role in CML and tumor sensitivity in tyrosine kinase inhibitors are not known. CXCR9-11 are CXCR3/CCR5 chemokine ligands and linked to T-helper 1 responses, which are important to antitumor immunity. The tumor microenvironment of CML is immunosuppressive, as cytotoxic T-cell function is extremely suppressed [21]. AF4 encodes a serine/proline-rich protein and is involved in transcriptional activation [22]. In addition, AF4 plays an important role in oncogenesis of acute lymphoblastic leukemia because the mixed lineage leukemia (MLL) gene located on 11q23 fuses to the AF4 genes and makes a chimera MLL/AF4 fusion protein [23–25]. MLL/AF4 chimera contributes to enhancing the hematopoietic repopulating cell function and clonogeneic potential that plays a crucial role in leukemogenesis [26]. Interestingly, a three-way translocation involving 4q21, t(4;15;17)(q21;q22;q21) was also reported in acute promyelocytic leukemia (APL) [27]. Unfortunately, we have no available samples to test these imporant genes in this case.

Regarding the clinical characteristics of previous reports, six patients were <60 years old, and the sex ratio (male to female) was about 3:1. Unfortunately, treatment was not described in most cases, although patients with variant Ph translocations treated with imatinib mesylate had a similar prognosis to those without variant Ph translocations [4, 5]. However, trisomy 8, a second Ph chromosome, isochromosome 17q, or trisomy 19 seem to have a negative impact on survival and progression [6]. There is no evidence showing that selection of second-generation TKIs benefit these patients, but Tirrò *et al.* reported that second-generation TKIs may be effective in these patients [28]. Similarly, our patient was treated with a second-generation TKI that was associated with a rapid decrease in BCR-ABL/ABL transcripts and achieved MR4.5 at 17 months after initial treatment. Our case is the first successful report treated with TKIs for patients with t(4;9;22)(q21;q34;q11.2). However, because this particular translocation has not been previously described, we cannot comment on its impact on clinical course. Further studies will be required to determine the effectiveness of TKIs in CML with this particular variant translocation.

While mechanisms promoting the generation of the variant translocations observed in CML are poorly understood, two different mechanisms have been suggested. A one-step mechanism in which chromosome breakage occurs on three different chromosomes simultaneously and leads to a three-way translocation has been suggested. Others have proposed a two-step mechanism in which a standard two-way t(9;22) translocation is followed by subsequent translocations involving an additional chromosome [29, 30].

## Conclusion

We report the first case of a complex three-way Ph chromosome variant t(4;9;22)(q21;q34;q11.2) successfully treated with a second-generation TKI. The pathological role of this complex three-way Ph chromosome variant warrants further study.

## Data Availability

All data generated or analyzed during this study are included in this published article. Additional information is available from the corresponding author upon reasonable request.

## Abbreviations

CML: Chronic myeloid leukemia
Ph: Philadelphia
CP: Chronic phase
TKIs: Tyrosine kinase inhibitors
WBC: White blood cell
CMR: Complete molecular response
PTPN13: Protein tyrosine-phosphatase non-receptor type 13
CXCL: Chemokine (C-X-C motif) ligand
RAP1GDS1: RAP1 GTPase-GDP dissociation stimulator 1
AF4: ALL1-fused gene from chromosome 4
MLL: Mixed lineage leukemia
